# Electroacupuncture regulates gut microbiota to reduce depressive-like behavior in rats

**DOI:** 10.3389/fmicb.2024.1327630

**Published:** 2024-03-27

**Authors:** Junying Wang, Haohan Zhu, Xingke Song, Jun Zhao, Jianliang Zhang, Jinling Zhang, Shaoyuan Li, Peijing Rong

**Affiliations:** Department of Physiology, Institute of Acupuncture and Moxibustion, China Academy of Chinese Medical Sciences, Beijing, China

**Keywords:** electroacupuncture, depression, gut microbiota, neurotransmitter, rats

## Abstract

**Background and objectives:**

Growing studies show that gut microbiota is closely associated with depression. Acupuncture treatment could regulate the gut microbiota of many diseases. Here, we aim to observe the effect of electroacupuncture (EA) on gut microbiota in rats that showed depressive-like behavior.

**Materials and methods:**

The rats were randomly divided into normal group, chronic unpredictable mild stress model (CUMS) group, CUMS + electroacupuncture (EA) group, and CUMS + sham-electroacupuncture (Sham) group. The CUMS+EA rats were treated with EA stimulation at bilateral Zusanli (ST36) and Tianshu (ST25) acupoints for 2 weeks (0.7 mA, 2/100 Hz, 30 min/day). The rats in the sham EA group were treated with the same conditions without inserting needles and electrical stimulation. Behavioral tests were conducted by forced swimming test (FST), open field test (OFT), and sucrose preference test (SPT) to assess depression-like behavior in rats. The relative abundance of intestinal bacteria in rat feces was detected by 16S rRNA analysis. The expression of calcitonin-gene-related peptide (CGRP), vasoactive intestinal peptide (VIP), somatostatin (SST), and adrenocorticotropic hormone (ACTH) in serum was detected by ELISA kit, and VIP, CGRP, and SST in the colon were detected by qRT-PCR and Western blot.

**Results:**

Chronic unpredictable mild stress model rats exhibited depressive-like behaviors and had differential abundance vs. control rats. CUMS significantly decreased the relative abundance of Bifidobacterium and Streptococcus at the genus level, CGRP in plasma (*p* < 0.05), and significantly increased the intestine propulsion rate, the mRNA and protein expression of VIP, SST, and mRNA in the colon, and ATCH in plasma (*p* < 0.05). EA rats with microbial profiles were distinct from CUMS rats. EA markedly reduced the depressive-like behaviors, significantly increased the intestine propulsion rate, the relative abundance of Bacteroidetes, Proteobacteria, and Actinobacteria at the phylum level, Bifidobacterium and Streptococcus at the genus level, and VIP and CGRP in plasma (*p* < 0.05), and significantly decreased Firmicutes, the ratio of Firmicutes to Bacteroidetes at the phylum level, ACTH and SST in plasma, and SST mRNA in the colon (*p* < 0.05).

**Conclusion:**

The antidepressant effect of EA at ST36 and ST25 is related to regulating intestinal flora and the neurotransmitter system. Our study suggests that EA contributes to the improvement of depression, and gut microbiota may be one of the mechanisms of EA effect.

## 1 Introduction

Depression is a common mental illness worldwide that affects more than 300 million people of all ages, according to the World Health Organization (WHO) (GBD 2017, 2018). Antidepressant medication as tricyclic antidepressants (TCAs) and selective serotonin reuptake inhibitors (SSRIs) has also produced significant side effects that patients do not tolerate (Malhi and Mann, 2018). Therefore, antidepressant non-pharmacological therapy has gotten more and more attention.

Acupuncture, as a traditional Chinese Medicine, has a good therapeutic effect on treating depression for thousands of years. Accumulating evidence also showed that acupuncture or electroacupuncture (EA) could be a non-pharmacological therapy for treating different kinds of depression, such as depression-related insomnia (Dong et al., 2017), postpartum depression (Li et al., 2018), depression in methamphetamine (MA) addicts during abstinence, and promote rehabilitation of patients (Zeng et al., 2018), post-stroke depression (PSD) (Zhang et al., 2019), and perimenopausal depression (Xiao et al., 2019). Acupuncture combined with antidepressant medications shows a better therapeutic effect than SSRI therapy alone (Chan et al., 2015). A meta-analysis showed that acupuncture may decrease Hamilton Rating Scale (HAMD) for Depression. At the same time, no significant effects on clinical response, Edinburgh Postnatal Depression Scale (EPDS), and serum estradiol levels were observed (Li et al., 2019). However, the underlying mechanism of effect of acupuncture for depression is not precise.

A growing body of research evidence suggests that depression has strongly associated with gut microbiome disorder (Winter et al., 2018). The microbiota-depleted rats that received the fecal microbiota from the depressed patients showed depressive-like behavior and physiological feature characteristic (Kelly et al., 2016). The bidirectional regulation between the brain and the gut microbiome mediates by the enteric nervous system–vagus nerve–central nervous system (Carabotti et al., 2015), the hypothalamic–pituitary–adrenal (HPA) axis (Misiak et al., 2020), Neuroendocrine-Immune (Liu et al., 2020), and neuroendocrine (Zalar et al., 2018). There are many ways of brain–gut interaction: probiotics may provide a useful novel treatment method for neuropathological disorders and/or as an adjunct treatment for psychiatric disorders (Vlainić et al., 2016; Paiva et al., 2020).

The emerging field of research focused on the development of preventive and therapeutic interventions for depression targeting the gut microbiota (Dash et al., 2015). Psychobiotics containing a variety of Lactobacillus and Bifidobacterium have been shown to reduce anxiety, improve mood, and enhance cognitive function in both healthy people and patient populations (Butler et al., 2019). *Bifidobacterium adolescentis* NK98 and *Lactobacillus reuteri* NK33 may relieve colitis and depression by improving intestinal microecological dysbiosis (Han et al., 2020). *Bifidobacterium breve* CCFM1025 also showed significant antidepressant like effect in mice (Tian et al., 2020). Another study demonstrated that probiotics improve mental flexibility and change gut microbiota at the same time in healthy elderly (Kim et al., 2021). The above data suggest that some prebiotics have a positive effect on the central nervous system (CNS) and may therefore play an important role in regulating depression.

The ancient classic “Lingshu meridians” clearly points out that the stomach meridian of Foot-Yangming could treat mental diseases. The study on the regularity of acupoint selection and the compatibility in the treatment of postpartum depression showed that the main acupoints were selected including Zusanli (ST36) (Zheng et al., 2023), but the mechanism is unclear. The aim of present study was to observe whether the antidepressant effect of EA was regulated by intestinal flora, so we selected the acupoints on the stomach meridian of Foot-Yangming. ST36 and Tianshu (ST 25) have been proven to regulate gut microbiota. Mild moxibustion at bilateral ST 25 and ST36 significantly increased the relative DNA abundances of Bifidobacterium and Lactobacillus but decreased that of *Escherichia coli* in rats with irritable bowel syndrome (IBS) (Bao et al., 2019). Mild moxibustion at the bilateral Shangjuxu (ST37) and ST25 significantly increased alpha diversity, downregulated the abundance of Bacteroides and Prevotella, and upregulated the abundance of Lactobacillus for the irritable bowel syndrome (IBS) rats (Wang et al., 2018). ST 25 and ST36 are on the stomach meridian of Foot-Yangming, which mainly treats gastrointestinal and mental disorders. Therefore, in the present study, ST36 that can be used as an anti-depression acupoint on the stomach channel of Zyangming and ST 25, a common point that can regulate intestinal flora, were selected to observe the regulatory effect of EA on the intestinal tract in depressed rats induced by chronic unpredictable mild stress (CUMS). It was speculated that the gut microbiota may play an important role in the process of the antidepressant effect of EA on depressed rats.

## 2 Materials and methods

### 2.1 Animal preparation

All protocols were carried out according to the National Institutes of Health Guide for the Care and Use of Laboratory Animals and approved by the Institutional Animal Care and Use Committee of China Academy of Chinese Medical Sciences (D2019-02-11-2).

**Figure 1:**
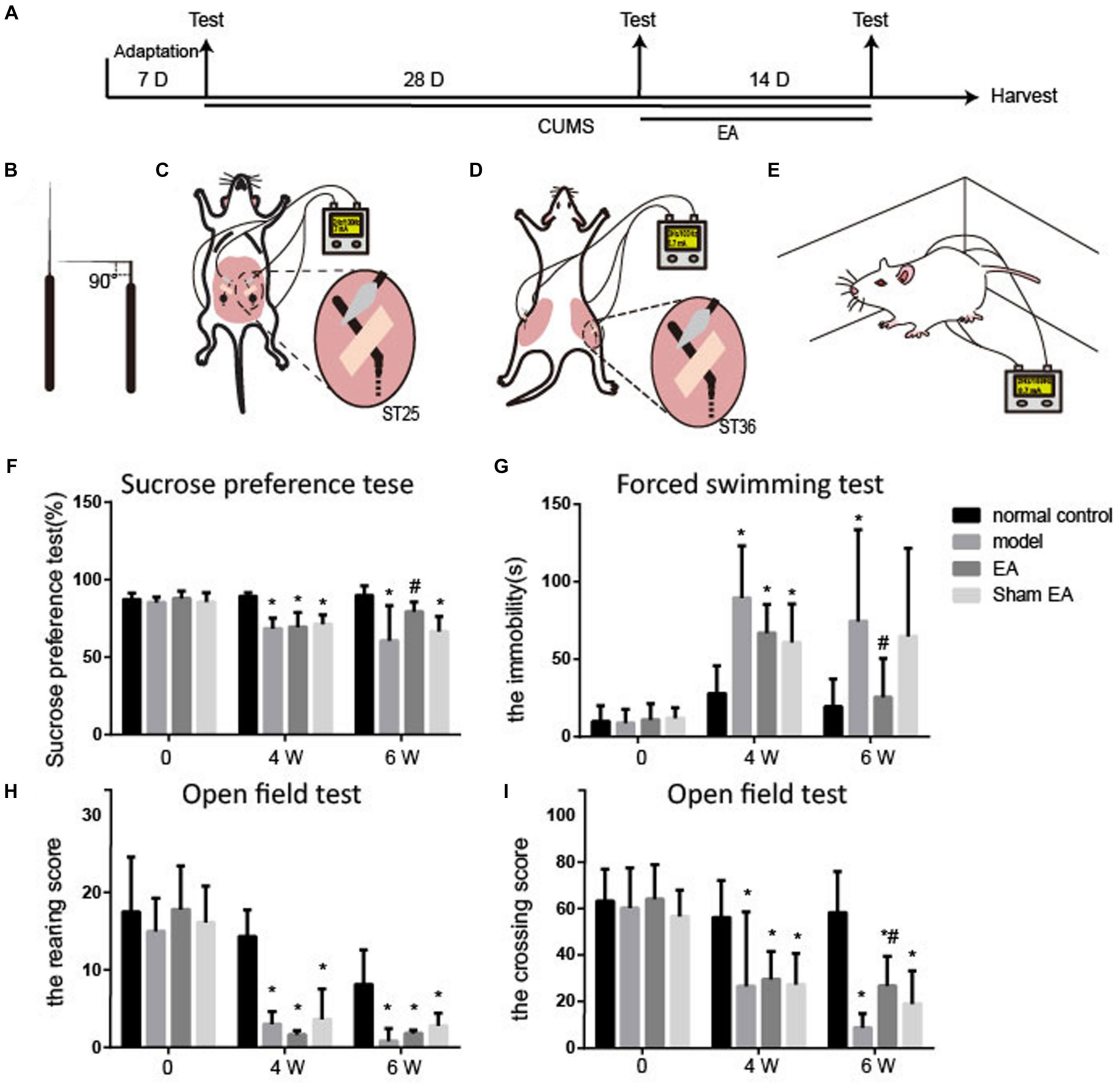
EA reduced depressive-like behaviors in CUMS rats (*n* = 6). **(A)** The experimental procedures of CUMS, EA treatment, and behavioral measurements. **(B–E)** Schematic drawing of the process of EA treatment. **(B)** The acupuncture was fold. **(C)** The bent acupuncture needle was inserted and fixed at the location of ST25. **(D)** The bent acupuncture needle was inserted and fixed at the location of st36. **(E)** The free moving rats during the EA treatment. **(F)** Sucrose preference test. **(G)** Forced swimming test. **(H,I)** Open field test. The effect of EA at ST 25 and ST36 on sucrose preference test, open field test, and forced swimming test in CUMS rats (mean ± SD).^*^p < 0.01, vs. the normal control group; ^#^*p* < 0.05, vs. the model group.

Male Sprague–Dawley rats (180–200 g, *n* = 24) were obtained from the Laboratory Animal Center of the Academy of Military Medical Sciences [license number: SCXK (Jing) 2014–0013]. All rats were subjected to a 12–12-h light–dark cycle with controlled temperature (22 ± 2°C) and humidity environment (50%), allowing them to freely get food and water. The health condition of animals was inspected daily. After 1 week of adaptation, the rats were randomly divided into four groups (*n* = 6/group): normal, chronic unpredictable mild stress model (CUMS, model), CUMS + electroacupuncture (EA), and CUMS + sham-electroacupuncture (Sham EA). Behavioral tests, including forced swimming test (FST), open field test (OFT), and sucrose preference test (SPT), were administered before and after 28 days of the CUMS procedure and after 2 weeks of EA treatment (Figure 1A).

### 2.2 The chronic unpredictable mild stress procedure

The CUMS model in the present experiment was a validated model of depression and adapted from other researchers (Zhang et al., 2016) with a minor modification. Each animal in CUMS group and CUMS+EA and CUMS+ sham group was socially isolated in a separate cage and administered the following different stressors for 42 days: swimming in ice water for 10 min (at 0°C); food deprivation for 24 h; electric foot shock for 30 times (2 mA for 10 s every 50 s); clipping tails for 1 min (1 cm from the base of the tail); water deprivation for 24 h; housing in a wet padding for 24 h (200 mL of water per cage); 45°cage tilted for 14 h; light–dark cycle reversal for 24 h; and physical restraint for 3 h. Rats received one of these stimulus each day, and the same stimulus was not applied consecutively over 2 days. The normal group is not disturbed, except for necessary procedures such as regular cage cleaning.

### 2.3 EA treatment

The rats from the EA group underwent EA stimulation at bilateral ST36 and ST25 acupoints. The hair of the location of acupoints of ST36 and ST25 was removed off for the fixation of acupuncture needles. The sterile acupuncture needles (0.3 mm × 13.0 mm, Suzhou Medical Appliance Factory, Jiangsu, China) at 5 mm were fold into 90° (Figure 1B) and inserted into the ST36 and ST25 acupoints at the depth of 5 mm, according to the location of each acupoint. The upper bent part of the sterile needle handles were fixed onto the surface of the rats’ skin with an adhesive tape (Figures 1C,D). The needle handles were connected with an electrical stimulator (Hanshi-100A; Nanjing Jisheng Medical Technology Company, Nanjing, China) and stimulated at an alternating frequency of 2/100 Hz for 30 min and an intensity of 0.7 mA each day for 2 weeks, while the rats were awake and free moving in a cage (Figure 1E). The rats were observed during the process of entire EA treatment. The acupuncture needle rarely falls off under this situation. However, if the acupuncture needle does fall off during the process of electroacupuncture treatment, the acupuncture needle would be re-inserted into the acupoints and re-fix with adhesive tape again to ensure EA treatment for 30 min of each experimental animal. The rats from the sham EA group were given the same conditions without a needle inserted and electrical stimulation.

ST36 is located approximately 5 mm below the fibular and on the posterolateral side of the hind limb knee joint (Li, 2007); ST25 is located on 5 mm lateral to the intersection of upper 2/3 and the lower 1/3, connecting the xiphoid process with the upper edge of the pubic symphysis (Yu et al., 2016).

### 2.4 Assessment of depressive-like behavior

For all behavioral tests, rats were transported in their home cages to the testing room at least 1 day before testing to get acclimated to the testing environment. A video tracking system was used to record the behavior of the rats. The experimenters were blinded to group identity during the experiment and quantitative analyses.

### 2.5 Sucrose preference test

Before the SPT, the rats were trained to drink sucrose solution (1%) for 24 h and then were given one bottle of 1% sucrose solution and one bottle of pure water for another 24 h. Then, the rats were deprived of water for 24 h, following 1-h test that the each rat was given a bottle of 1% sucrose solution and a bottle of pure water. The two bottles were weighed and recorded before and after the testing. Sucrose preference was calculated by the following formula: sucrose preference (%) = sucrose intake/(sucrose intake + water intake) × 100%.

### 2.6 Open field test

The open field test (OFT) measured the rats’ exploratory behavior and general activity. The rats were placed in the center of the bottom of the apparatus (100 cm × 100 cm × 40 cm black chamber, with 25 equal-size squares marked by white lines) and freely explore the surrounding for 3 min. The numbers of crossing (squares crossed by limb) and rearing (hind limb standing times) were counted during the 3-min period. Before each test, the inner wall and bottom surface of the apparatus were wiped with 75% alcohol to avoid any residual information from the previous rats.

### 2.7 Forced swimming test

Forced swimming test is one of the most commonly used assays for evaluating the depressive-like behavior in small animals such as rodents to reflect behavioral despair (Yankelevitch-Yahav et al., 2015). Rats were placed in a pressure environment filled with water (a cylinder container with 45 cm height, 25 cm diameter) at 23 ± 1°C and forced to swim for 15 min. After 24 h, the rats were placed in the same water for 5 min. The swimming, struggling, and immobility time were recorded.

### 2.8 Gastric emptying test

After fasting for 24 h, the rats were gavaged and fed with a liquid test meal (2 mL) that contained a non-absorbable marker phenolic red (50 mg/dL). The rats were deep anesthesia with pentobarbital sodium (35 mg/kg, i.p.) and then sacrificed. After ligating the cardia and pylorus, the stomach was removed, and gastric contents were rinsed with distilled water. The distilled water was added to 20 mL into the gastric solution, and 20 mL of 0.5 mol/L NAOH was added and mixed. After 1 h, 5 mL supernatant was collected and added 0.5 mL trichloroacetic acid (20%). The mixed liquid was centrifuged for 10 min with a radius of 3,500 r/min; then, the absorbance value (OD) was detected at 560 nm. The standard OD of phenol red was generated with 20 mL phenolic red (50 mg/dL) at 560 nm. Gastric emptying rate (%) was calculated as (1 − measured phenol red OD/standard phenol red OD) × 100%.

### 2.9 Detection of small intestine propulsion rate

After the rats were sacrificed, the whole small intestine was removed and straightly put on ice to observe the length of the small intestine, dyed red with phenol red. If the end of the dyed red of the small intestine becomes purple after dropping a small amount of 0.5 mol/L NAOH solution, it is where phenol red arrives. Then, a small amount of NAOH solution was added before and after the purplish red area to determine the farthest location of phenol red reached. The distances from the pylorus to ileocecal length (A) and from the pylorus to phenol red stained red end (a) were measured. The small intestine propulsion rate was (%) = a/A × 100%.

### 2.10 Sample collection

Blood and colon were collected after the rats were sacrificed. The serum was centrifuged and separated at 3,000 rpm for 10 min at 4°C and then stored at −20°C. Colon and hypothalamus samples were collected immediately and stored under −80°C.

### 2.11 16S rRNA analysis of fecal samples

The fecal collected samples were stored into 1.5 mL tubes under −80°C. The total DNA of fecal samples was extracted using the E.Z.N.A. Stool DNA Kit (Omega Bio-Tek, Inc., United States). The V3-V4 region of the 16S rRNA gene of bacteria was amplified from the fecal DNA extracts by modified universal bacterial primer pairs 515F (5’-TCGTCG GCAGCGTCAGATG TGTATAAGAGACAGGT GCCAGCMGCCGCGGTAA-3′) and 806R (5’-GTCTCGTGGGCT CGGAGATGTGTATAAGAGACAGGGACTACHVGGGTWTCTAA T-3′) with Illumina adaptor overhang sequences. The amplification program consisted of one pre-denaturation cycle for 5 min at 95°C, 28 denaturation cycles for 45 s at 95°C, annealing for the 50s at 55°C, extension for 45 s at 72°C, and finally 1 extension cycle for 10 min at 72°C. After that, the PCR products were purified by Agencourt AMPure XP (Beckman Coulter, Inc., United States) and sequenced using an Illumina MiSeq sequencing platform (Illumina, San Diego, CA, United States).

Use QIIME (v1.8.0) software to divide the data into different samples. The sequences were merged and overlapped relationship using Pear (v0.9.6) software to by the overlapping relationship. Then, the sequences with lengths less than 230 bp were removed by Vsearch (v2.7.1) software, and the de-chimera sequences were compared by the uchime method according to the Gold Database. Afterward, operational taxonomic units (OTUs) clustering of high-quality sequences using the UPARSE algorithm of Vsearch (v2.7.1) sofware. The BLAST algorithm was used to compare the OTU representative sequence with the Silva138 data. According to OTU and its abundance results, the QIIME (v1.8.0) software and R (v3.6.0) software were used to calculate and plot the α-diversity index, respectively. The histogram of species composition was analyzed by R (v3.6.0) software. The β-diversity distance matrix was calculated by the QIIME (v1.8.0) software, and the PCoA and NMDS were analyzed by the R (v3.6.0) software on the basis of the distance matrix. Sequence data supporting the results of this study are available at the National Center for Biotechnology Information with the main entry code PRJNA924098.

### 2.12 Western blot analysis

The total protein was extracted from the tissue by protein lysate containing protease and phosphatase inhibitors (Roche, Shanghai, China) using a tissue homogenizer. An equal amount of protein in each sample was subjected to 5 or 8% sodium dodecyl sulfate-polyacrylamide gel electrophoresis (SDS-PAGE) at 90/160 V for 60 min and then electro-transferred to polyvinylidene difluoride (PVDF) membrane (Millipore Corporation, Billerica, MA, United States) at 90 mA for 150 min. The 5% bovine serum albumin (BSA, Amresco, Solon, OH, United States) solution was used to block at room temperature for 60 min. Then, the membranes were incubated with first antibody vasoactive intestinal peptide (VIP) (1: 500; ab8556, Abcam, Cambridge, United Kingdom), calcitonin-gene-related peptide (CGRP) (1:500; ab47027, Abcam, Cambridge, United Kingdom), and GAPDH (1: 20,000, TDY042, Tiandeyue Biological Technology Company, Beijing, China) overnight at 4°C. After incubated with secondary antibody [donkey anti-goat IgG 1:10,000 diluted or donkey anti-rabbit Immunoglobulin (Ig)G 1:20,000 diluted; Jackson Immuno Research Laboratories, West Grove, PA, United States] at room temperature for 1 h, the membranes were developed by an enhanced chemiluminescence (ECL) detection system. The TotalLab Quant analysis software (TotalLab Limited, Newcastle upon Tyne, United Kingdom) was used to scan and quantify the blots for densitometric analyses. The primer sequences are shown in Table 1.

**Table 1:** Primer sequences.

Primer	Sequence(5′–3′)	Length (bp)
Sst forward	CGAGCCCAACCAGACAGAG	225
Sst reverse	TTTGGAGGAGAGGGATCAGAG	
VIP forward	TGATGTGTCCAGAAATGCCA	123
VIP reverse	CTACTGCTGATTCGTTTGCC	
GAPDH forward	CCTTCCGTGTTCCTACCCC	131
GAPDH reverse	GCCCAGGATGCCCTTTAGTG	

### 2.13 Enzyme-linked immunosorbent assay

The concentrations of vasoactive intestinal peptide (VIP), CGRP, somatostatin (SS), and ACTH in serum were detected by ELISA kit (R&D Systems, United States) as the protocol provided by the manufacturer’s instructions.

### 2.14 Quantitative real-time PCR

The total RNA was extracted from tissue with reagent (Life Technologies, Carlsbad, California) and reverse-transcribed to cDNA by Prime Script TM Reagent Kit with gDNA Eraser (Takara Bio, Shiga, Japan). Quantitative real-time (QRT) PCR was performed using cDNA as template by the QRT-PCR detection systems (ABI7500, Applied Biosystems, United States). The qPCR reaction system conditions were 95°C 30 s; 95°C 5 s, 60°C 10 s, and 72°C 15 s, with a total of 40 cycles. Using GAPDH as internal reference, the relative expression of mRNA of target genes was calculated by the 2^−ΔΔCt^ method.

### 2.15 Statistical analysis

Measurement data are expressed as the mean ± SD, and SPSS17.0 software (SPSS, Chicago, IL, United States) was used for data analysis. Data between groups were compared using one-way ANOVA and the least significant difference test. LSD test was used for post-test when variance was equal, and Games-Howell test was used for post-test when variance was not equal. *p* < 0.05 indicated that the difference was statistically significant.

## 3 Results

### 3.1 EA significantly alleviated the depressive-like behaviors

As shown in Figure 1, no differences in sucrose preference, the immobility time, and the rearing or the crossing score were observed between the four groups before the CUMS procedure.

Compared with the normal control group, the rearing and crossing score, and sucrose preference were observably decreased (*p* < 0.05), and the immobility time markedly increased (*p* < 0.05, Figure 1B) in the model, EA, and sham groups after CUMS modeling for 4 weeks. It is suggested that the rats showed depressive-like behaviors after CUMS modeling.

The sucrose preference and the crossing score were upregulated in the EA group (*p* < 0.05, Figures 1D,E), and the sham group also exhibited an increase in sucrose preference and the crossing score, but there were no significances (*p* > 0.05) after 2 weeks of treatment. The immobility time in EA or sham group was observably increased, compared to the model group (*p* < 0.05, Figure 1C). The rearing score was not changed after EA treatment. EA observably alleviated the CUMS-induced depressive-like behaviors.

### 3.2 EA reduced the intestine propulsion rate but not the gastric emptying rate

**Figure 2:**
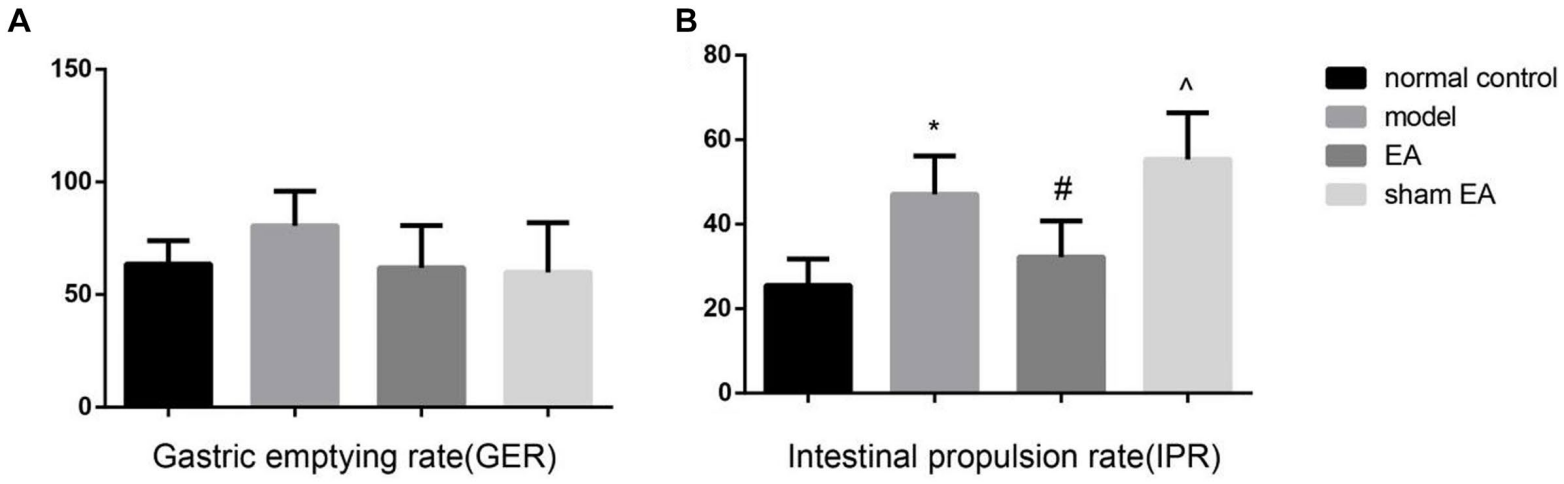
The gastric emptying rate (GER) and intestine propulsion rate (IPR) in the different groups (*n* = 6). **(A)** The GER in the different groups. **(B)** The IPR in the different groups. ^*^p < 0.01, vs the normal control group; ^#^*p* < 0.05, vs the model group, ^^^*p* < 0.01, vs the EA group.

The gastric emptying rate has no difference between the different groups (*p* > 0.05, Figure 2A). Compared with normal control group, the intestine propulsion rate was markedly increased in the model group (*p* < 0.05). EA significantly decreased the intestinal propulsion rate (*p* < 0.05, Figure 2B), and the sham EA had little effect on the intestinal propulsion rate (*p* > 0.05).

### 3.3 EA had little effect on the intestinal microbial diversity

**Figure 3:**
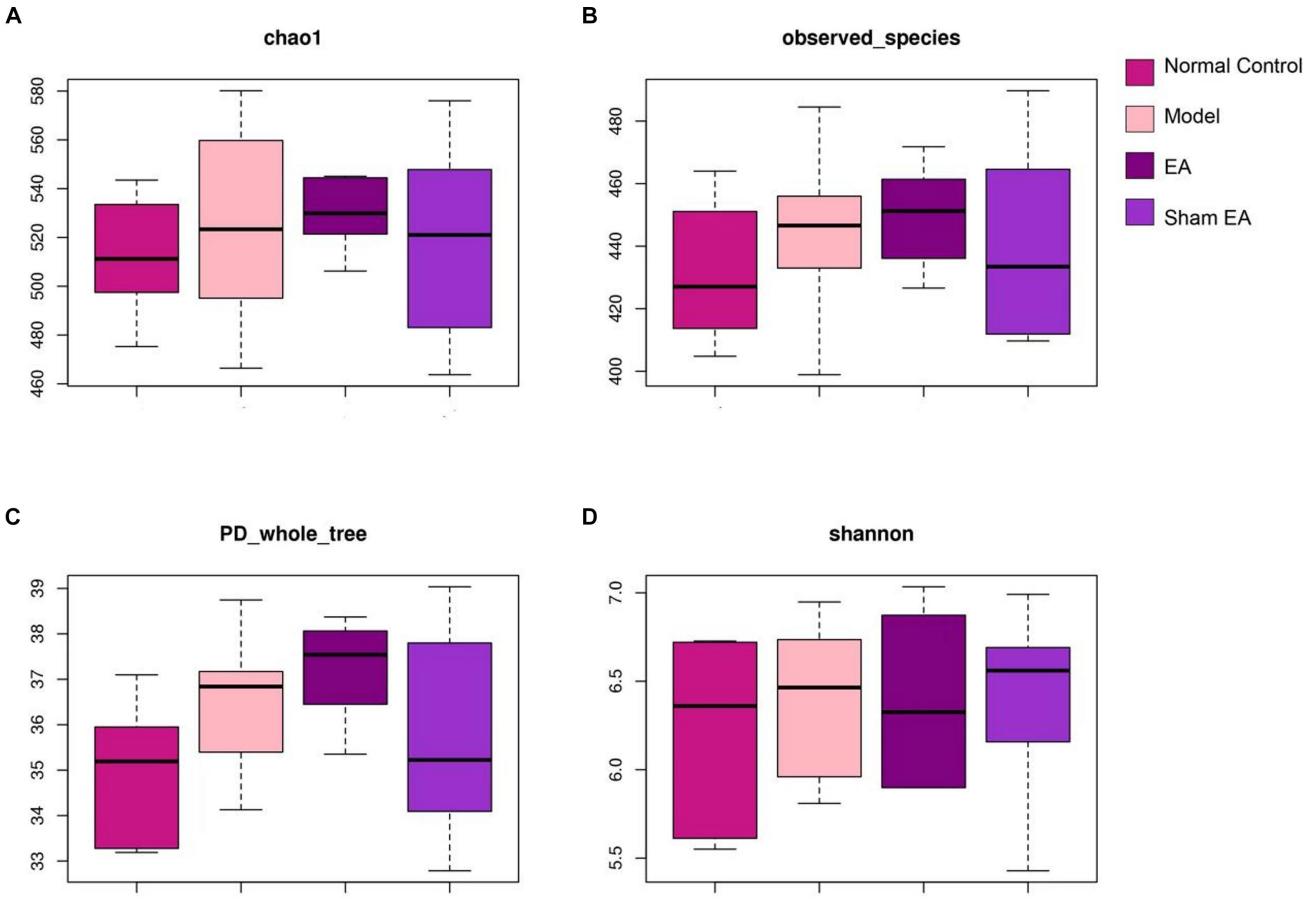
16S rRNA gene sequencing reveals that EA had no effect on the α-diversity of intestinal microflora including **(A)** chao1 index, **(B)** observed species index, **(C)** PD whole tree and **(D)** shannon index (*n* = 6).

Single sample diversity analysis reflected the abundance and diversity of the microbial community. Alpha-diversity metrics refer to the species richness, diversity, and evenness under the local uniform biological environment, which was also known as intrabiological diversity. To evaluate the α-diversity of intestinal microflora, the microbial community richness was characterized by the Chao1 index and observed species index, the diversity of the microbial community was characterized by the Shannon index, and the evolution-based diversity of microbial communities was characterized by the PD-whole-tree index. There were no obvious differences among the four groups according to alpha diversity (*p* > 0.05, Figures 3A–D). It is indicated that CUMS model and EA have little effect on intestinal microbial diversity.

### 3.4 EA changed the microbial flora

**Figure 4:**
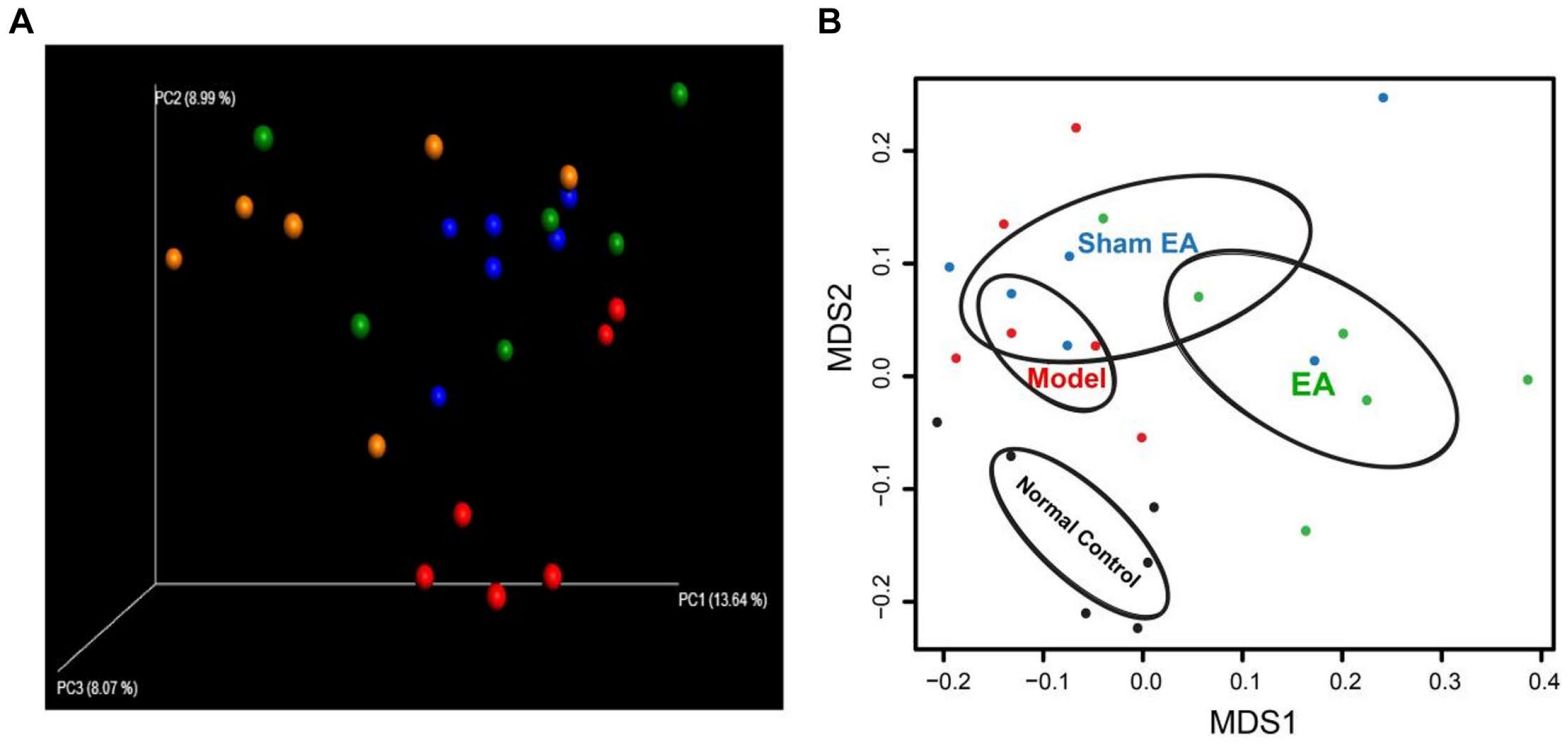
16S rRNA gene sequencing reveals changes to microbial flora in EA rats (*n* = 6). **(A)** Three-dimensional principal coordinate analysis (PCoA) of unweighted UniFrac distances showed an obvious difference in gut microbiota composition between EA rats and model rats. Red: Normal Control, Blue: Model, Orange: EA, and Green: Sham EA. **(B)** NMDS based on weighted UniFrac distance between samples of rats in different groups. Black: Normal Control, Red: Model, Green: EA, and Blue: Sham EA.

We used non-metric multidimensional scaling (NMDS) analyses and principal coordinate analysis (PCoA), which examine relationships between ecologic communities, such as microbial communities, to determine whether those OTUs identified to differentiating rats in a different groups (Figure 4). The higher the coincidence rate between the two groups, the more similar the composition of the gut microbiota between the two groups, and the lower the coincidence rate between the two groups, the farther the distance between them. The results showed that the composition of the intestinal microflora was different between the two groups of rat. PCoA was used to assess whether there are clusters or groupings in the data. PCoA allows to observe the similarities and differences between samples. PCoA indicated the presence of four groups (Figure 4A). Based on the PCoA, the samples were labeled as control (control rats with microbial profiles distinct from model rats and EA rats), model (model rats with microbial profiles distinct from EA rats), Sham EA (sham EA rats with similar microbial composition as EA rats and model rats), and EA (EA rats with microbial profiles distinct from control rats and model rats; Figure 4A). NMDS analysis identified that the model rats have differential abundance vs. control rats and the samples were completely divided into two distinct groups, with EA rats vs. model rats (Figure 4B).

### 3.5 EA regulated the relative abundance of gut microbiota at the phylum level

**Figure 5:**
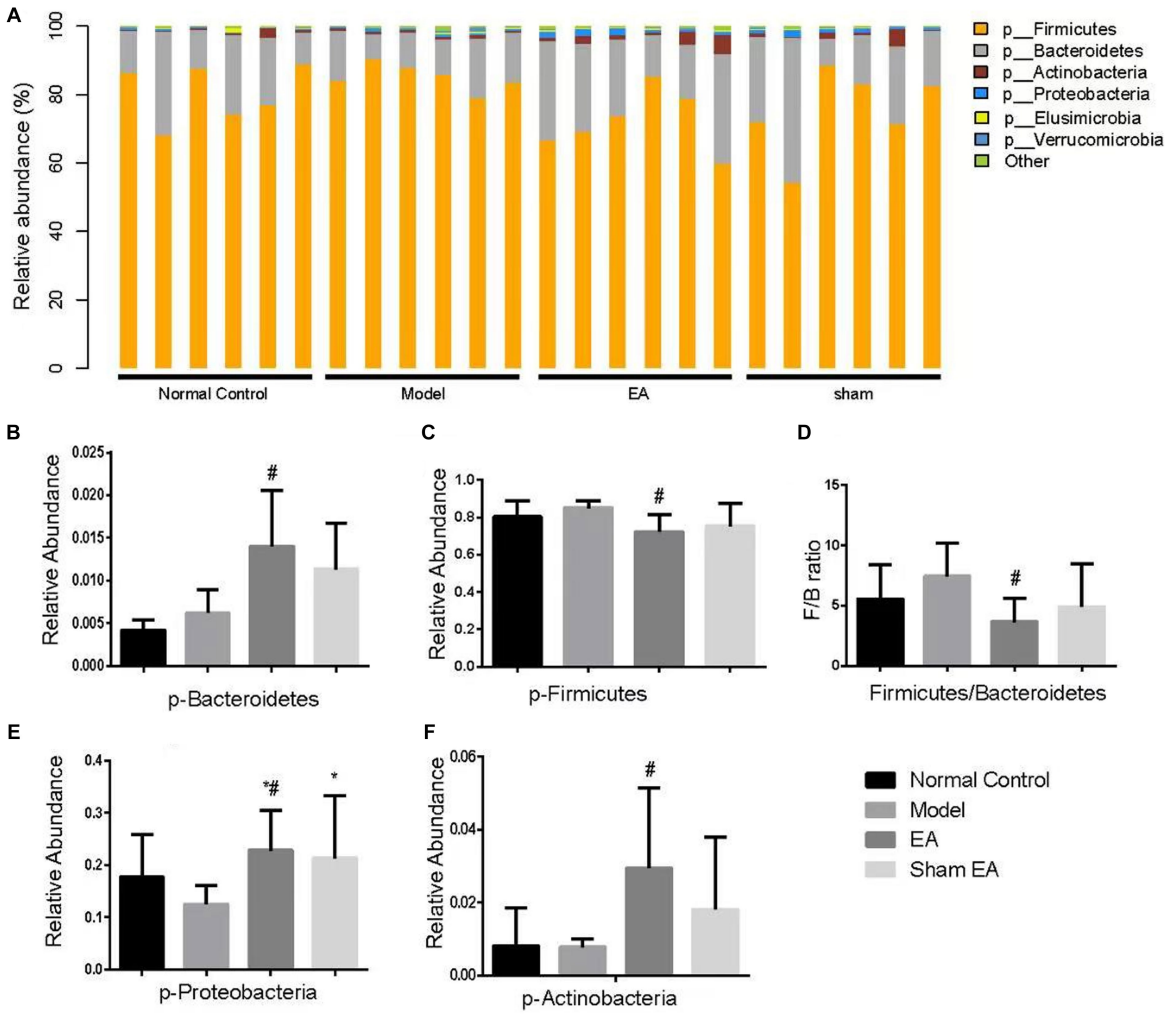
Comparison of gut microbiota composition at the phylum level in rats from each group (*n* = 6). **(A)** Relative abundances of the phylum present in samples from different groups of rats. **(B–F)** The relative abundances of Bacteroidetes, Firmicutes, Proteobacteria, and Actinobacteria were significantly changed in EA rats as compared with model rats. ^*^p < 0.01, vs. the normal control group; ^#^*p* < 0.05, vs. the model group, and ^^^*p* < 0.01, vs. the EA group.

The relative abundance of phylum shows differences in samples from different groups (Figure 5A). Compared with the normal control group, the relative abundance of Bacteroidetes, Firmicutes, and others has no significant difference at the phylum level in the model group (*p* > 0.05, Figures 5B–F). Compared with the model group, the relative abundance of Bacteroidetes, Proteobacteria, and Actinobacteria significantly increased (*p* < 0.05, Figures 5B,E,F), Firmicutes significantly decreased (*p* < 0.05, Figure 5C), and the ratio of Firmicutes to Bacteroidetes significantly reduced (*p* < 0.05, Figure 5D).

### 3.6 EA regulated the relative abundance of gut microbiota at the genus level

**Figure 6:**
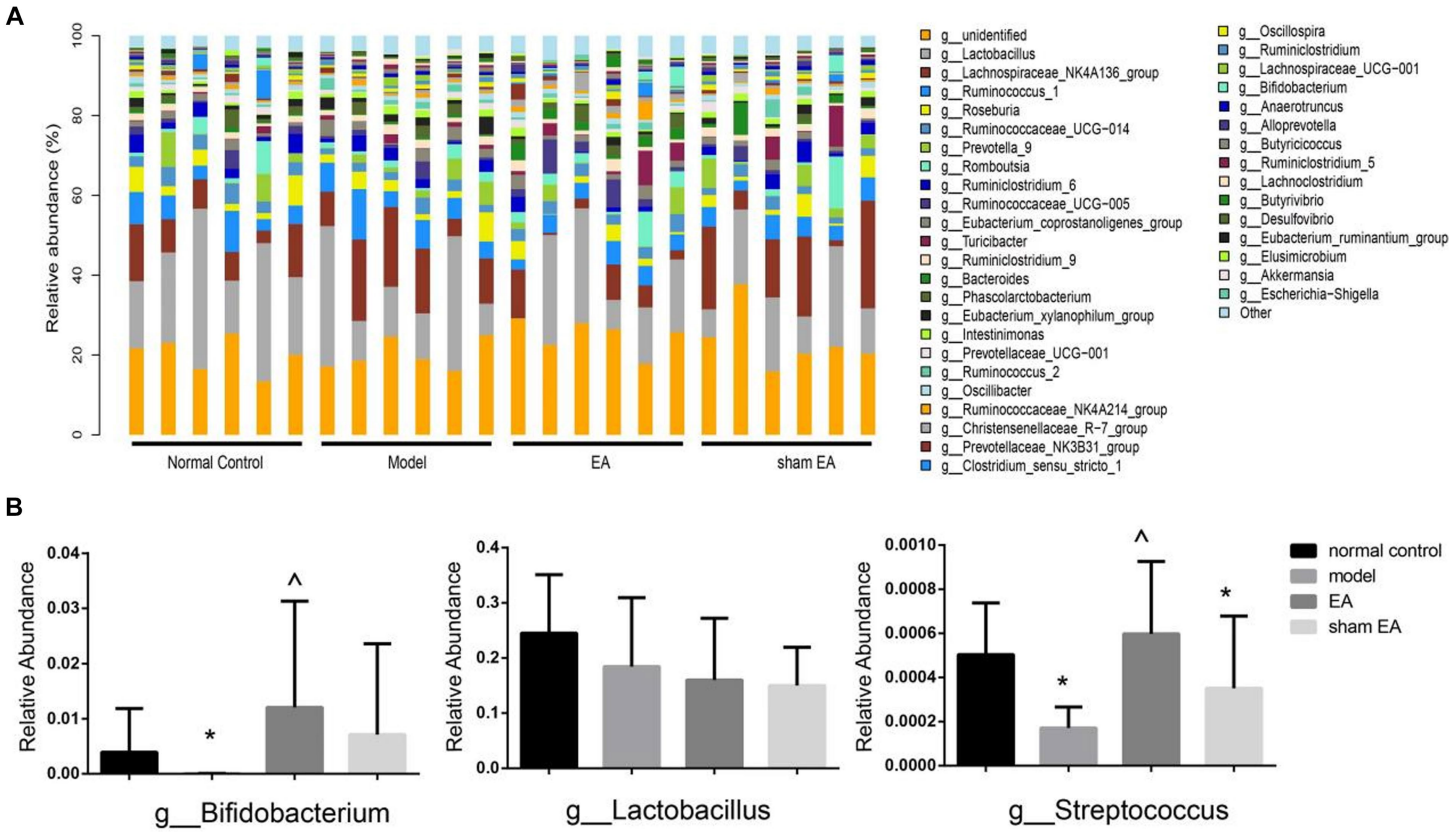
Comparison of gut microbiota composition at the genus level in rats from each group (*n* = 6). **(A)** Relative abundances of the genus present in samples from different group rats. **(B)** The relative abundances of Bifidobacterium, Lactobacillus, and Streptococcus in a different group. ^*^p < 0.01, vs. the normal control group; ^#^*p* < 0.05, vs. the model group, and ^^^*p* < 0.01, vs. the EA group.

The relative abundance of the genus shows differences in samples from different groups (Figure 6A). Compared with the normal control group, the relative abundance of Bifidobacterium and Streptococcus was significantly decreased at the genus level in the model group (*P* < 0.05, Figure 6B). The relative abundance of Bifidobacterium and Streptococcus significantly increased after EA treatment (*p* < 0.05, Figure 6B). There was no significant difference in the relative abundance of Lactobacillus at the genus level among different groups (*p* > 0.05, Figure 6B).

### 3.7 EA regulated the expression of neuroendocrine in the colon and plasma

**Figure 7:**
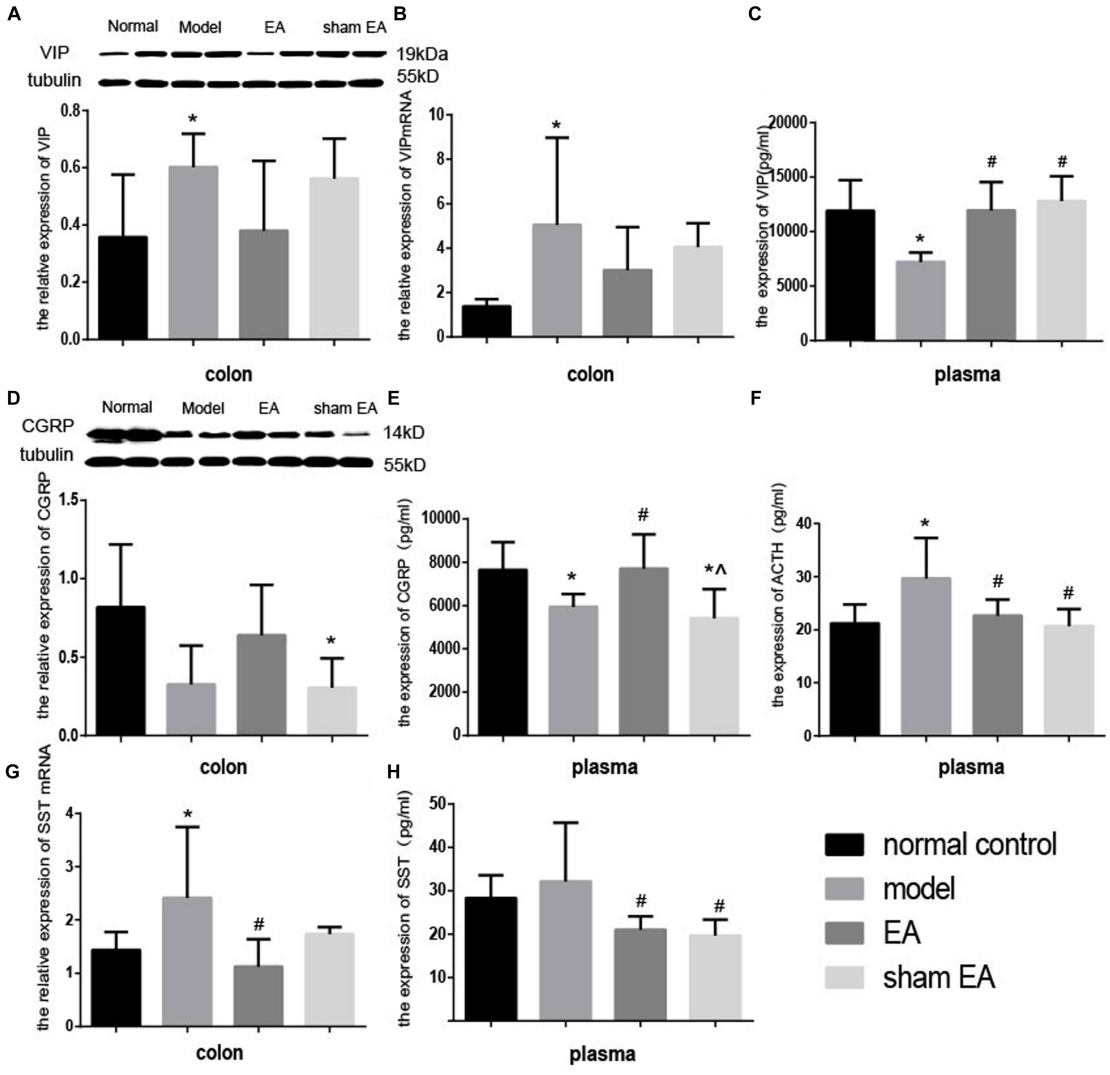
Effect of EA on the expression of VIP CGRP, ACTH, and SST in colon tissues and plasma (*n* = 6). ^*^p < 0.01, vs. the normal control group; ^#^*p* < 0.05, vs. the model group, ^^^*p* < 0.01, vs. the EA group.

Compared with the normal control group, the expressions of VIP protein and VIP mRNA increased significantly in the model group (*p* < 0.05, Figures 7A,B) in the colon and the expression of VIP decreased markedly in plasma (*p* < 0.05, Figure 7C). Compared with the model group, the expressions of VIP protein and VIP mRNA decreased in the EA group (*p* > 0.05, Figures 7A,B) in the colon, and the expression of VIP significantly increased in the EA and sham EA group in plasma (*p* < 0.05, Figure 7C).

The expressions of CGRP in the colon and plasma decreased slightly (*p* > 0.05, Figure 7D) and markedly (*p* < 0.05, Figure 7E) in the model group compared to the normal control group, respectively. The expression of the CGRP significantly increased (*p* < 0.05) in the colon and plasma. Compared with the EA group, the expression of CGRP in plasma decreased significantly (*p* < 0.05) in the sham EA group, compared to the model group. The expressions of CGRP in the colon and plasma after sham EA intervention were still lower than that of the normal control group (*p* < 0.05).

Compared to the normal control group, after CUMS modeling, the expression of ACTH increased significantly (*p* < 0.05, Figure 7F) in plasma. The expression of ACTH in EA and sham EA groups was lower than in the model group (*p* < 0.05, Figure 7F).

Compared with the normal control group, the relative expression of SST mRNA in the colon and the expression of SST in plasma increased decreased markedly (*p* < 0.05, Figure 7G) and slightly (*p* < 0.05, Figure 7H) in the model group. The relative expression of SS mRNA in the colon and the expression of SS significantly decreased (*p* < 0.05, Figures 7G,H) in the EA group, compared with the model group.

## 4 Discussion

Acupuncture, a highly favored complementary therapy, is widely recognized for its potential benefits in treating depression. A meta-analysis of randomized trials revealed that acupuncture had a positive impact on the HAMD score, particularly when combined with antidepressant medications. This combination therapy not only improved the severity of depression and sleep quality but also demonstrated an early onset of action. Furthermore, the treatment was found to be safe and well-tolerated throughout the initial 6-week period (Chan et al., 2015; Dong et al., 2017). For postpartum depression and depression, acupuncture treatment could significantly reduce HAMD scores (Li et al., 2019). Accumulating evidence suggests that acupuncture affects depression, but its under mechanism is still unclear, which limits it to guide future clinical directions (Smith et al., 2018). In the present study, we conducted a series of research on the effect of acupuncture on depressive rats, using measures related to depressive disorder, including observation of behavior, neuropeptides, and alterations. After 4 weeks of CUMS modeling, the rats showed significant depressive behaviors, and EA treatment of ST36-ST25 for 2 weeks improved the depressive behaviors as the sucrose preference, immobility time, and crossing score increased significantly. However, there was no such effect in the sham EA group.

In recent years, an increasing amount of research has provided support for the notion that imbalances in gut microbiota and microbiota–gut–brain dysfunction may contribute to the development of major depression (Liang et al., 2018). The gut microbiota may significantly impact the development of depression through the brain–gut–microbiota axis, affecting both neural and immune systems (Chang et al., 2022). Using clinical samples, a notable disparity was observed in the gut microbiota compositions between individuals suffering from major depressive disorder (MDD) and healthy controls. MDD patients displayed substantial alterations in the relative abundance of phyla—Firmicutes, Actinobacteria, and Bacteroidetes (Zheng et al., 2016). In the rat model of depression induced by chronic variable stress (CVS), significant alterations in the gut microbiota were observed, particularly at the phylum and genus levels (Yu et al., 2017). In our findings, the genus-level analysis revealed a slight increase in the relative abundance of phyla—Bacteroidetes and the Firmicutes/Bacteroidetes ratio following CUMS modeling.

Electroacupuncture had a substantial impact on the gut microbiota composition in obese abdominal rats. This impact was primarily characterized by a decrease in the Firmicutes/Bacteroidetes ratio and an increase in Prevotella abundance (Wang et al., 2019). Acupuncture at acupoints GB34 and ST36 changed the relative abundance of 18 genera in Parkinson’s disease (PD) rats: Butyricimonas, Holdemania, Frisingicoccus, Gracilibacter, Phocea, and Aestuariispira exhibited substantial correlations with both anxiety and motor functions (Jang et al., 2020), but there is little research to observe the effect of acupuncture or electroacupuncture on the gut microbiota in depressive rats. In this study, EA treatment had little effect on the diversity of intestinal microflora but had some effect on the relative abundance in CUMS rats. The application of EA treatment led to a noteworthy rise in the proportion of Bacteroidetes, Proteobacteria, and Actinobacteria, while decreasing the ratio of Firmicutes/Bacteroidetes.

The gut microbiota and the central nervous system communicate bidirectionally via the microbiota–gut–brain axis (Du et al., 2020). Therefore, the gut microbiota can be targeted for both prevention and treatment of depression; this includes the possibility of probiotic supplementation (Ansari et al., 2020; Paiva et al., 2020). Microbiome-based treatments such as probiotics are suggested for depression to cultivate helpful bacteria in the gut (Mörkl et al., 2020). Thus, we also observed the changes in the probiotics after EA treatment for depressive rats. The relative abundance of g-Bifidobacterium and g-Streptococcus significantly decreased after CUMS modeling, and EA treatment markedly increased the relative abundance of g-Bifidobacterium and g-Streptococcus. A 16-week, double-blind, randomized, placebo-controlled trial showed that probiotic supplements containing *Lactobacillus helveticus* and Bifidobacterium long might be effective in alleviating symptoms of depression (Wallace et al., 2020). Hence, it is proposed that EA’s antidepressant impact correlates with the adjustment of the proportionate occurrence of probiotics, including g-Streptococcus and g-Bifidobacterium that mitigate depressive behaviors.

Depression causes intestinal flora disorders and is associated with neuroendocrine disorders. Neuropeptides such as substance P, CGRP, SST, and adrenocorticotropin-releasing factor released by specialized cells within the gastrointestinal tract are likely to play an important role in the bidirectional signaling between the gut and brain (Holzer and Farzi, 2014). Chronic stress can cause injury to the duodenum and reduce VIP levels in the plasma and duodenum (Huang et al., 2007). Chronic mild stress can increase SST expression levels in plasma (Faron-Górecka et al., 2016). Anxiety-depressive states could result in altered SS and VIP secretion, which in turn affects gastrointestinal motility and function (Han, 2013). These changes in neuropeptides may be caused by altered regulation of gut microbes. Fecal microbiota from patients with depression increased hippocampal ACTH (Liu et al., 2020). Probiotics can increase the relative abundance of Lactobacillus and Bifidobacterium and decrease the level of serum ACTH (Li et al., 2021). Triple viable Bifidobacterium plus smecta reduced the serum levels of VIP and improved the clinical symptoms with diarrhea (Zhao, 2015). Those peptides are strong modulation of neuroinflammation, have also been found to exert direct antimicrobial effects, and they thought to be in involved in the bidirectional relationship between the gut microbiota and the host (including gut-brain axis) (Aresti Sanz and EI, 2019). Also, these peptides are thought to have direct antimicrobial effects, modulate neuroinflammation, and are therefore able to orchestrate changes in the gut microbial community and participate in the bidirectional relationship between the gut and the brain (Holzer and Farzi, 2014). In the present research, EA treatment downregulated the increased SST mRNA in colon and SST in plasma, and ACTH in the plasma which may be induced by the increased expression of probiotics (Bifidobacterium). The mice showed depression-like behavior and decreased CGRP levels, and intracerebroventricular CGRP administration has anti-depressive effect (Hashikawa-Hobara et al., 2015). This is consistent with our results that EA treatment upregulated the protein expression of CGRP in the colon and CGRP in plasma.

Gastric emptying rate and intestinal propulsion rate are important manifestations of gastrointestinal motility. Germ-free mouse studies have demonstrated that the presence of a gastrointestinal microbiota is crucial for maintaining normal gastrointestinal motility (Dimidi et al., 2017). Specifically, in the absence of gut microbiota, there is an increase in gastric emptying and gut transit time compared to wild-type mice (Abrams and Bishop, 1967; Iwai et al., 1973). Multi-probiotics could significantly improve the major depressive disorder (MDD) patients’ gastrointestinal functions (Tian et al., 2023). Our research findings showed that EA significantly reduces the intestine propulsion rate and slightly reduces the gastric emptying rate which may be directly induced in part by the gut microbiota including Bifidobacterium. Neuropeptides including VIP, SST, and ACTH also regulate gastrointestinal motility. VIP is a gastrointestinal hormone that can inhibit gastrointestinal motility and gastric emptying and reduce gastrointestinal motility and excitability. CGRP can regulate intestinal sensory function, inhibit gastric acid secretion, and reduce gastric emptying. EA treatment increased the expression of VIP in plasma, the protein expression of CGRP in the colon, and CGRP in plasma that may induce the intestine propulsion rate and the gastric emptying.

Our study proves that EA treatment is a promising non-pharmacological treatment method to alleviate depressive symptoms, provides new evidence for the selection of acupoints for clinical treatment of depression, and provides reference for improving the efficacy of clinical treatment of depression. It is hoped that it can open up ideas for clinicians.

## 5 Limitations

The present study has identified a correlation between the antidepressant properties of EA treatment and its modulation of the intestinal microbiota in a rodent model of depression. However, to elucidate the underlying neuroanatomical mechanisms that underpin this relationship, additional investigations are warranted. Further research endeavors should delve into the intricate interplay between the central nervous system, the gut–brain axis, and the microbiota–gut–brain axis, aiming to unravel the intricate neural circuitry and signaling pathways involved. Such studies may shed light on the specific regions, neural networks, and neurotransmitter systems that are implicated in the antidepressant effects of EA and its influence on gut microbial composition.

## 6 Conclusion

In our study, we observe that EA at ST36 and ST25 could significantly alleviate depression-like behavior induced by CUMS in rats. This antidepressant effect may be related to the regulation of gut microbiota and neurotransmitters.

## Data availability statement

The datasets presented in this study can be found in online repositories. The names of the repository/repositories and accession number(s) can be found in the article/supplementary material.

## Ethics statement

The animal study was approved by the Institutional Animal Care and Use Committee of China Academy of Chinese Medical Sciences (D2019-02-11-2). The study was conducted in accordance with the local legislation and institutional requirements.

## Author contributions

JW: Writing – original draft. HZ: Data curation, Writing – review & editing. XS: Data curation, Writing – review & editing. JuZ: Data curation, Writing – review & editing. JiaZ: Data curation, Writing – review & editing. JinZ: Writing – review & editing. SL: Writing – review & editing. PR: Writing – review & editing.

## References

[ref1] Abrams G. D. Bishop J. E. (1967). Effect of the normal microbial flora on gastrointestinal motility. Proc. Soc. Exp. Biol. Med. 126, 301–304. doi: 10.3181/00379727-126-32430, PMID: 6066182

[ref2] Ansari F. Pourjafar H. Tabrizi A. Homayouni A. (2020). The effects of probiotics and prebiotics on mental disorders: a review on depression, anxiety, Alzheimer, and autism Spectrum disorders. Curr. Pharm. Biotechnol. 21, 555–565. doi: 10.2174/1389201021666200107113812, PMID: 31914909

[ref9001] Aresti Sanz J. El Aidy S. (2019). Microbiota and gut neuropeptides: a dual action of antimicrobial activity and neuroimmune response. Psychopharmacology (Berl). 236: 1597–1609. doi: 10.1007/s00213-019-05224-0, PMID: 30997526 PMC6598950

[ref3] Bao C. H. Wang C. Y. Li G. N. Yan Y. L. Wang D. Jin X. M.. (2019). Effect of mild moxibustion on intestinal microbiota and NLRP6 inflammasome signaling in rats with post-inflammatory irritable bowel syndrome. World J. Gastroenterol. 25, 4696–4714. doi: 10.3748/wjg.v25.i32.4696, PMID: 31528095 PMC6718040

[ref4] Butler M. I. Sandhu K. Cryan J. F. Dinan T. G. (2019). From isoniazid to psychobiotics: the gut microbiome as a new antidepressant target. Br. J. Hosp. Med. 80, 139–145. doi: 10.12968/hmed.2019.80.3.139 30860919

[ref5] Carabotti M. Scirocco A. Antonietta M. A. Severi C. (2015). The gut-brain axis: interactions between enteric microbiota, central and enteric nervous systems. Ann. Gastroenterol. 28, 203–209. PMID: 25830558 PMC4367209

[ref6] Chan Y. Y. Lo W. Y. Yang S. N. Chen Y. H. Lin J. G. (2015). The benefit of combined acupuncture and antidepressant medication for depression: a systematic review and meta-analysis. J. Affect. Disord. 176, 106–117. doi: 10.1016/j.jad.2015.01.048, PMID: 25704563

[ref7] Chang L. Wei Y. Hashimoto K. (2022). Brain-gut-microbiota axis in depression: a historical overview and future directions. Brain Res. Bull. 182, 44–56. doi: 10.1016/j.brainresbull.2022.02.004, PMID: 35151796

[ref8] Dash S. Clarke G. Berk M. Jacka F. N. (2015). The gut microbiome and diet in psychiatry: focus on depression. Curr. Opin. Psychiatry 28, 1–6. doi: 10.1097/YCO.0000000000000117 25415497

[ref9] Dimidi E. Christodoulides S. Scott S. M. Whelan K. (2017). Mechanisms of action of probiotics and the gastrointestinal microbiota on gut motility and constipation. Adv. Nutr. 8, 484–494. doi: 10.3945/an.116.014407, PMID: 28507013 PMC5421123

[ref10] Dong B. Chen Z. Yin X. Li D. Ma J. Yin P.. (2017). The efficacy of acupuncture for treating depression-related insomnia compared with a control group: a systematic review and Meta-analysis. Biomed. Res. Int. 2017, 9614810–9614811. doi: 10.1155/2017/9614810, PMID: 28286776 PMC5329663

[ref11] Du Y. Gao X. R. Peng L. Ge J. F. (2020). Crosstalk between the microbiota-gut-brain axis and depression. Heliyon 6:e04097. doi: 10.1016/j.heliyon.2020.e04097, PMID: 32529075 PMC7276434

[ref12] Faron-Górecka A. Kuśmider M. Kolasa M. Żurawek D. Szafran-Pilch K. Gruca P.. (2016). Chronic mild stress alters the somatostatin receptors in the rat brain. Psychopharmacology 233, 255–266. doi: 10.1007/s00213-015-4103-y, PMID: 26462807 PMC4700104

[ref13] GBD 2017 (2018). Disease and injury incidence and prevalence collaborators. Global, regional, and national incidence, prevalence, and years lived with disability for 354 diseases and injuries for 195 countries and territories, 1990-2017: a systematic analysis for the global burden of disease study 2017. Lancet 392, 1789–1858. doi: 10.1016/S0140-6736(18)32279-7, PMID: 30496104 PMC6227754

[ref14] Han B. (2013). Correlation between gastrointestinal hormones and anxiety-depressive states in irritable bowel syndrome. Exp. Ther. Med. 6, 715–720. doi: 10.3892/etm.2013.1211 24137253 PMC3786850

[ref15] Han S. K. Kim J. K. Joo M. K. Lee K. E. Han S. W. Kim D. H. (2020). *Lactobacillus reuteri* NK33 and *Bifidobacterium adolescentis* NK98 alleviate *Escherichia coli*-induced depression and gut Dysbiosis in mice. J. Microbiol. Biotechnol. 30, 1222–1226. doi: 10.4014/jmb.2002.02058, PMID: 32347078 PMC9728327

[ref16] Hashikawa-Hobara N. Ogawa T. Sakamoto Y. Matsuo Y. Ogawa M. Zamami Y.. (2015). Calcitonin gene-related peptide pre-administration acts as a novel antidepressant in stressed mice. Sci. Rep. 5:12559. doi: 10.1038/srep12559, PMID: 26251188 PMC4528222

[ref17] Holzer P. Farzi A. (2014). Neuropeptides and the microbiota-gut-brain axis. Adv. Exp. Med. Biol. 817, 195–219. doi: 10.1007/978-1-4939-0897-4_9, PMID: 24997035 PMC4359909

[ref18] Huang Y. L. Yu J. P. Wang G. H. Chen Z. H. Wang Q. Xiao L. (2007). Effect of fluoxetine on depression-induced changes in the expression of vasoactive intestinal polypeptide and corticotrophin releasing factor in rat duodenum. World J. Gastroenterol. 13, 6060–6065. doi: 10.3748/wjg.v13.45.6060, PMID: 18023100 PMC4250891

[ref19] Iwai H. Ishihara Y. Yamanaka J. Ito T. (1973). Effects of bacterial flora on cecal size and transit rate of intestinal contents in mice. Jpn. J. Exp. Med. 43, 297–305. PMID: 4580917

[ref20] Jang J. H. Yeom M. J. Ahn S. Oh J. Y. Ji S. Kim T. H.. (2020). Acupuncture inhibits neuroinflammation and gut microbial dysbiosis in a mouse model of Parkinson's disease. Brain Behav. Immun. 89, 641–655. doi: 10.1016/j.bbi.2020.08.015, PMID: 32827699

[ref21] Kelly J. R. Borre Y. O' Brien C. Patterson E. el Aidy S. Deane J.. (2016). Transferring the blues: depression-associated gut microbiota induces neurobehavioural changes in the rat. J. Psychiatr. Res. 82, 109–118. doi: 10.1016/j.jpsychires.2016.07.019, PMID: 27491067

[ref22] Kim C. S. Cha L. Sim M. Jung S. Chun W. Y. Baik H. W.. (2021). Probiotic supplementation improves cognitive function and mood with changes in gut microbiota in community-dwelling older adults: a randomized, double-blind, placebo-controlled, multicenter trial. J. Gerontol. A Biol. Sci. Med. Sci. 76, 32–40. doi: 10.1093/gerona/glaa090, PMID: 32300799 PMC7861012

[ref23] Li ZR (2007). Experimental Acupuncture. Beijing: Chinese Press of Traditional Chinese Medicine, 273–274

[ref24] Li Q. Li L. Niu X. Tang C. Wang H. Gao J.. (2021). Probiotics alleviate depressive behavior in chronic unpredictable mild stress rat models by remodeling intestinal flora. Neuroreport 32, 686–693. doi: 10.1097/WNR.0000000000001637, PMID: 33913925

[ref25] Li W. Yin P. Lao L. Xu S. (2019). Effectiveness of acupuncture used for the Management of Postpartum Depression: a systematic review and Meta-analysis. Biomed. Res. Int. 2019:6597503. doi: 10.1155/2019/6597503, PMID: 31016194 PMC6446093

[ref26] Li S. Zhong W. Peng W. Jiang G. (2018). Effectiveness of acupuncture in postpartum depression: a systematic review and meta-analysis. Acupunct. Med. 36, 295–301. doi: 10.1136/acupmed-2017-011530, PMID: 29907576

[ref27] Liang S. Wu X. Hu X. Wang T. Jin F. (2018). Recognizing depression from the microbiota^−^Gut^−^Brain Axis. Int. J. Mol. Sci. 19:1592. doi: 10.3390/ijms19061592, PMID: 29843470 PMC6032096

[ref28] Liu S. Guo R. Liu F. Yuan Q. Yu Y. Ren F. (2020). Gut microbiota regulates depression-like behavior in rats through the neuroendocrine-immune-mitochondrial pathway. Neuropsychiatr. Dis. Treat. 16, 859–869. doi: 10.2147/NDT.S243551, PMID: 32280227 PMC7127849

[ref29] Malhi G. S. Mann J. J. (2018). Depression. Lancet 392, 2299–2312. doi: 10.1016/S0140-6736(18)31948-2 30396512

[ref30] Misiak B. Loniewski I. Marlicz W. Frydecka D. Szulc A. Rudzki L.. (2020). The HPA axis dysregulation in severe mental illness: can we shift the blame to gut microbiota? Prog. Neuro-Psychopharmacol. Biol. Psychiatry 102:109951. doi: 10.1016/j.pnpbp.2020.109951, PMID: 32335265

[ref31] Mörkl S. Butler M. I. Holl A. Cryan J. F. Dinan T. G. (2020). Probiotics and the microbiota-gut-brain Axis: focus on psychiatry. Curr. Nutr. Rep. 9, 171–182. doi: 10.1007/s13668-020-00313-5, PMID: 32406013 PMC7398953

[ref32] Paiva I. H. R. Duarte-Silva E. Peixoto C. A. (2020). The role of prebiotics in cognition, anxiety, and depression. Eur. Neuropsychopharmacol. 34, 1–18. doi: 10.1016/j.euroneuro.2020.03.006, PMID: 32241688

[ref33] Smith C. A. Armour M. Lee M. S. Wang L. Q. Hay P. J. (2018). Acupuncture for depression. Cochrane Database Syst. Rev. 2018:CD004046. doi: 10.1002/14651858.CD004046.pub4, PMID: 29502347 PMC6494180

[ref34] Tian P. O'Riordan K. J. Lee Y. K. Wang G. Zhao J. Zhang H.. (2020). Towards a psychobiotic therapy for depression: *Bifidobacterium breve* CCFM1025 reverses chronic stress-induced depressive symptoms and gut microbial abnormalities in mice. Neurobiol. Stress 12:100216. doi: 10.1016/j.ynstr.2020.100216, PMID: 32258258 PMC7109524

[ref35] Tian P. Zou R. Wang L. Chen Y. Qian X. Zhao J.. (2023). Multi-probiotics ameliorate major depressive disorder and accompanying gastrointestinal syndromes via serotonergic system regulation. J. Adv. Res. 45, 117–125. doi: 10.1016/j.jare.2022.05.003, PMID: 35618633 PMC10006521

[ref36] Vlainić J. V. Šuran J. Vlainić T. Vukorep A. L. (2016). Probiotics as an adjuvant therapy in major depressive disorder. Curr. Neuropharmacol. 14, 952–958. doi: 10.2174/1570159x14666160526120928, PMID: 27226112 PMC5333591

[ref37] Wallace C. J. K. Foster J. A. Soares C. N. Milev R. V. (2020). The effects of probiotics on symptoms of depression: protocol for a double-blind randomized placebo-controlled trial. Neuropsychobiology 79, 108–116. doi: 10.1159/000496406 30759442

[ref38] Wang X. Qi Q. Wang Y. Wu H. Jin X. Yao H.. (2018). Gut microbiota was modulated by moxibustion stimulation in rats with irritable bowel syndrome. Chin. Med. 13:63. doi: 10.1186/s13020-018-0220-y, PMID: 30574173 PMC6299671

[ref39] Wang H. Wang Q. Liang C. Su M. Wang X. Li H.. (2019). Acupuncture regulating gut microbiota in abdominal obese rats induced by high-fat diet. Evid. Based Complement. Alternat. Med. 2019, 4958294–4958312. doi: 10.1155/2019/4958294, PMID: 31275411 PMC6582896

[ref40] Winter G. Hart R. A. Charlesworth R. P. G. Sharpley C. F. (2018). Gut microbiome and depression: what we know and what we need to know. Rev. Neurosci. 29, 629–643. doi: 10.1515/revneuro-2017-0072, PMID: 29397391

[ref41] Xiao X. Zhang J. Jin Y. Wang Y. Zhang Q. (2019). Acupuncture for perimenopausal depression: a protocol for a systematic review and meta-analysis. Medicine (Baltimore) 98:e14073. doi: 10.1097/MD.0000000000014073, PMID: 30633212 PMC6336580

[ref42] Yankelevitch-Yahav R. Franko M. Huly A. Doron R. (2015). The forced swim test as a model of depressive-like behavior. J. Vis. Exp. 97:52587. doi: 10.3791/52587, PMID: 25867960 PMC4401172

[ref43] Yu M. Jia H. Zhou C. Yang Y. Zhao Y. Yang M.. (2017). Variations in gut microbiota and fecal metabolic phenotype associated with depression by 16S rRNA gene sequencing and LC/MS-based metabolomics. J. Pharm. Biomed. Anal. 138, 231–239. doi: 10.1016/j.jpba.2017.02.008, PMID: 28219800

[ref44] Yu Z. Zhang N. Lu C. X. Pang T. T. Wang K. Y. Jiang J. F.. (2016). Electroacupuncture at ST25 inhibits jejunal motility: role of sympathetic pathways and TRPV1. World J. Gastroenterol. 22, 1834–1843. doi: 10.3748/wjg.v22.i5.1834, PMID: 26855542 PMC4724614

[ref45] Zalar B. Haslberger A. Peterlin B. (2018). The role of microbiota in depression—a brief review. Psychiatr. Danub. 30, 136–141. doi: 10.24869/psyd.2018.136, PMID: 29930222

[ref46] Zeng L. Tao Y. Hou W. Zong L. Yu L. (2018). Electro-acupuncture improves psychiatric symptoms, anxiety and depression in methamphetamine addicts during abstinence: a randomized controlled trial. Medicine (Baltimore) 97:e11905. doi: 10.1097/MD.0000000000011905, PMID: 30142795 PMC6112927

[ref47] Zhang X. Y. Li Y. X. Liu D. L. Zhang B. Y. Chen D. M. (2019). The effectiveness of acupuncture therapy in patients with post-stroke depression: an updated meta-analysis of randomized controlled trials. Medicine (Baltimore) 98:e15894. doi: 10.1097/MD.0000000000015894, PMID: 31145349 PMC6708961

[ref48] Zhang X. Song Y. Bao T. Yu M. Xu M. Guo Y.. (2016). Antidepressant-like effects of acupuncture involved the ERK signaling pathway in rats. BMC Complement. Altern. Med. 16:380. doi: 10.1186/s12906-016-1356-x, PMID: 27680977 PMC5041500

[ref49] Zhao L. F. (2015). The impact of triple viable Bifidobacterium capsule combined with Smecta on plasma VIP, NPY and mucosal 5-HT levels in children with diarrhea. Chin. J. Microecool. 27, 917–920. doi: 10.13381/j.cnki.cjm.201508013

[ref50] Zheng L. Liu X. Y. Lin L. Zhou D. F. Hu Y. Q. (2023). Analysis of regularity of acupoint selection and compatibility of acupuncture in the treatment of postpartum depression. Zhen Ci Yan Jiu 48, 305–310. doi: 10.13702/j.1000-0607.20211295, PMID: 36951085

[ref51] Zheng P. Zeng B. Zhou C. Liu M. Fang Z. Xu X.. (2016). Gut microbiome remodeling induces depressive-like behaviors through a pathway mediated by the host's metabolism. Mol. Psychiatry 21, 786–796. doi: 10.1038/mp.2016.44, PMID: 27067014

